# The genetic control of avascular area in mouse oxygen-induced retinopathy

**Published:** 2012-02-08

**Authors:** Bliss E. O’Bryhim, Jeff Radel, Stuart J. Macdonald, R.C. Andrew Symons

**Affiliations:** 1 Department of Ophthalmology, University of Kansas Medical Center, Kansas City, KS; 2 Molecular and Integrated Physiology, University of Kansas Medical Center, Kansas City, KS; 3 Occupational Therapy Education, University of Kansas Medical Center, Kansas City, KS; 4 Molecular Biosciences, University of Kansas, 1200 Sunnyside Avenue, Lawrence, KS

## Abstract

**Purpose:**

The C57BL/6ByJ and BALB/cByJ inbred strains of mice are, respectively, susceptible and resistant to oxygen-induced retinopathy (OIR). The purpose of this work was to investigate the genetic control of the retinal avascular area in mouse OIR using a mapping cross.

**Methods:**

The central retinal avascular area was measured on postnatal day 16 (P16) in C57BL/6ByJ, BALB/cByJ, 101 (C57BL/6ByJ x BALB/cByJ)F_2_, and 116 (BALB/cByJ x C57BL/6ByJ)F_2_ mice that had been subjected to the OIR protocol. A genome-wide scan was performed of selected albino and non-albino mice to determine quantitative trait loci associated with weight and avascular area.

**Results:**

C57BL/6ByJ mice had significantly larger avascular areas than BALB/cByJ ones. Albino mice of the F_2_ generation had smaller avascular areas than the non-albino mice. Genotyping was performed at 856 informative single nucleotide polymorphisms approximately evenly distributed across the genome from each of 85 selected F_2_ mice. Weight, sex, and the paternal grandmother were found to act as additive covariates associated with the avascular area on P16; mapping analyses that used a model incorporating these covariates found a quantitative trait locus on chromosome 7 related to avascular area. Mapping analyses that used a model that did not incorporate covariates found a quantitative trait locus on chromosome 9 related to avascular area. A quantitative trait locus for bodyweight on P16 was mapped to chromosome 5.

**Conclusions:**

The retinal avascular area in the mouse OIR model is under genetic control. Revascularization in OIR is related to the weight, strain of paternal grandmother, sex, and albinism. Our data support the existence of a quantitative trait locus on chromosome 5 that influences weight after exposure to hyperoxia, as well as quantitative trait loci on chromosomes 7 and 9 that modify susceptibility to OIR.

## Introduction

Retinopathy of prematurity (ROP) is a leading cause of vision loss in children in both developed and developing countries [1–3]. ROP is a complex disease where disruption of normal developmental angiogenesis initially leads to a persistent avascular phase, which is followed by a phase of pathological neovascularization. Known risk factors include the use of supplemental oxygen therapy, low birthweight, early gestational age at time of birth [4,5], and low serum levels of insulin-like growth factor 1 [6] and insulin-like growth factor binding protein 3 (IGFBP3) [7]. Several clinical investigations have found that rates of severe ROP differ between ethnic groups even after adjusting for socioeconomic factors such as access to healthcare, suggesting a heritable component to ROP development [2,8–10]. Polymorphisms in the Wnt pathway genes frizzled-4 (*FZD4*), low-density lipoprotein receptor-related protein 5 (*LRP5*), and Norrie disease protein (*NDP*) have been associated with individual cases of severe ROP [11–14]. However, as yet there are no genetic factors known to account for a major proportion of variability in the severity of ROP [10].

Oxygen induced retinopathy (OIR) in the mouse was developed as a model of ROP [15]. In the mouse OIR model, exposure to hyperoxia leads to central vaso-obliteration. When the mice are returned to a normoxic environment, the region of avascular retina becomes ischemic and creates a pro-angiogenic milieu that leads to retinal neovascularization [15,16]. The hyperoxic phase of OIR creates conditions somewhat similar to those created in the past by overuse of O_2_ in the treatment of premature infants. However, hyperoxia in the OIR model leads to obliteration of central retinal vessels, while ROP results from the failure of vascularization of the peripheral retina. The neovascular phases of OIR and ROP are broadly analogous. When mice that have been exposed to hyperoxia in the OIR model are returned to normoxia, the retina gradually revascularizes with vessels that appear anatomically normal. The initial stages of vascular regeneration are seen before pathological neovascular tufts become evident. The study of the revascularization phase of OIR may be pertinent to understanding retinal angiogenesis in the preproliferative phase of ROP. For example, a demonstration that exogenous IGFBP3 increased vessel regrowth in OIR was followed by the observation that serum IGFBP3 correlated with less severe ROP in humans [7].

Interstrain differences in response to OIR models have been investigated in both mice and rats. Van Wijngaarden [17] demonstrated that pigmentation status is associated with the retinal avascular area in crosses between the Fischer 344 and Dark Agouti rat strains. In crosses between these two strains, retinas from albino animals exposed to OIR were found to express lower levels of erythropoietin than retinas from pigmented animals. In further work [18], van Wijngaarden found that OIR-susceptible rat strains demonstrated reduced expression of angiogenesis-related genes in early retinal development than did OIR-resistant rats, but higher expression of these genes in the proliferative phase of OIR. Chan et al. [19] performed a similar study comparing gene expression in retinas of different mouse strains exposed to the OIR model. Dorrel et al. [20] have shown that the astrocytes in the retinas of C57BL/6J mice exposed to the OIR protocol, unlike the astrocytes of BALB/c mice, quickly degenerate after the mice are returned to normoxia. Although these strain-specific differences have been found, no previous study has employed a genome-wide approach to investigate genetic differences that modify OIR.

This study uses a known difference between BALB/cByJ and C57BL/6ByJ mice to investigate the genetics of control of the extent of avascular areas measured four days after returning to normoxia. These strains were used in a mapping cross designed to identify quantitative trait loci (QTL) that modify this phenotype. Since C57BL/6 mice exposed to the OIR model develop more severe neovascularization, and revascularize more slowly, than BALB/c mice—despite having similar vaso-obliterative responses [20]—the phenotype of the avascular area measured four days after returning to normoxia is likely to be largely a measure of the rate of vascular regrowth.

## Methods

### Animals

All experimental procedures were approved by the University of Kansas Medical Center Institutional Animal Care and Use Committee. BALB/cByJ and C57BL/6ByJ mice were purchased from Jackson Laboratories (Bar Harbor, ME). All mice were kept in a 12 h:12 h light-dark cycle with ambient room temperature between 19 °C and 22 °C. Mice were kept on a standard diet (8604 rodent diet; Harlan Laboratories, Indianapolis, IN), and breeding pairs were maintained on a higher-fat diet (8626 rodent diet; Harlan Laboratories), with chow and water available ad libitum.

**Figure 1:**
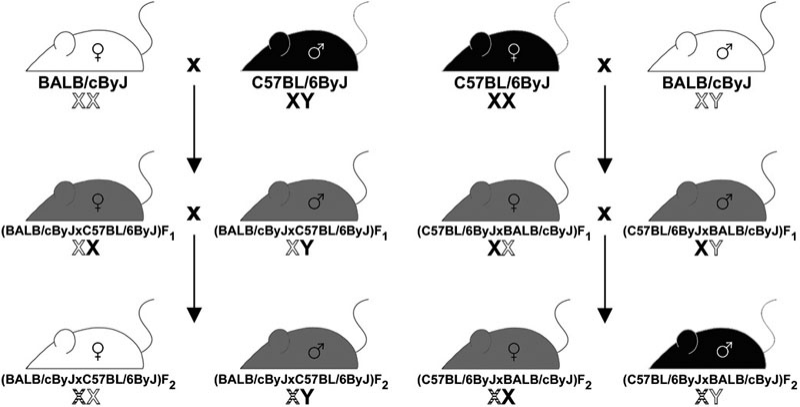
Illustration of the breeding scheme used in the mapping cross. The BALB/cByJ and C57BL/6ByJ parental strains are crossed in both directions. The resulting F_1_ mice are inbred through brother–sister mating to produce the F_2_ generation. This figure shows the manner in which the Y chromosomes and recombined (black and white-striped) and unrecombined (solid color) X chromosomes are passed through the generations of the cross. White chromosomes are inherited from the BALB/cByJ albino parent, and black chromosomes are inherited from the C57BL/6ByJ pigmented parent. The different coat colors shown in the F_2_ generation are for illustrative purposes only. The color of the F_2_ animals is unrelated to either gender or the direction of the cross.

An F_2_ intercross population was bred using reciprocal mating strategies (Figure 1). Phenotypes were obtained from 116 (BALB/cByJ x C57BL/6ByJ) F_2_ and 101 (C57BL/6ByJ x BALB/cByJ) F_2_ mice. Values were also obtained from BALB/cByJ and C57BL/6ByJ mice to confirm their differential susceptibility to OIR.

### Avascular areas in OIR

OIR was induced by exposing pups to 75% oxygen beginning on P7 and returning them to normoxia after 120 h. O_2_ levels in the chambers were continuously controlled using a ProOx portable monitor (Biospherix, Redfield, NY), which was calibrated monthly using room air and 100% O_2_. Retinas were analyzed 96 h after their return to hypoxia, since previous unpublished observations had suggested that at this time point the interstrain differences were greatest. This experimental plan allowed genetic modifiers of vascular regeneration to be investigated. On postnatal day 16 (P16), mice were weighed, anesthetized with a lethal dose of tribromoethanol delivered by intraperitoneal injection, perfused with high-molecular weight fluorescein isothiocyanate-labeled dextran (MW≥2,000,000; Sigma, St. Louis, MO) via the left ventricle to visualize patent vessels, and then enucleated. Tail samples were taken and stored at −20 °C, and the sex was documented.

Retinas were fixed overnight in 4% paraformaldehyde at 4 °C, dissected, and flatmounted using antifade medium (Southern Biotech). Immunofluorescent micrographic images of the retinas were taken using an SMZ 1500 microscope (Nikon, Tokyo, Japan) and MagnaFire Camera and software (Optronics, Goleta, CA). ImageJ software was used to manually trace and measure nonperfused areas. A mean avascular area was calculated for each mouse using data from left and right eyes.

### DNA preparation and genotype analysis

#### Genotyping strategy

Selective genotyping methods that decrease the cost of genotyping with only minor loss of information [21–23] were used. For the purpose of selecting the mice to genotype, the avascular area was adjusted for the weight of the animals to account for the effect of weight on the response to OIR. To do this, a correlation coefficient of −0.1743 mm^2^/g was determined using linear regression and multiplied by the weight to create an adjustment factor for each mouse. Since a significant difference was observed between the avascular areas of albino and non-albino mice, these groups were considered separately when choosing mice to genotype. A group of mice with the smallest adjusted avascular areas (11 albino and 32 non-albino mice) and a group with the largest adjusted areas (11 albino and 32 non-albino mice) were chosen for genotyping.

#### DNA preparation

DNA was extracted from 0.5 cm mouse tail samples using the DNEasy Blood and Tissue Kit (Qiagen, Valencia, CA); concentration and purity (absorbance ratio at 260/280 nm) were determined using a NanoDrop-1000 spectrophotometer (ThermoFisher Scientific, Waltham, MA). DNA samples were diluted to 25–150 ng/µl.

#### Genotype analysis

Genotyping services were provided by the Center for Inherited Disease Research (Johns Hopkins University, Baltimore, MD) using a standard linkage panel containing 856 informative autosomal and X chromosomal single nucleotide polymorphisms (SNPs; Mouse Medium Density Linkage Panel; Illumina, San Diego, CA). For each chromosome, except chromosome X, the most proximal marker genotyped was within 13.4 Mb of the centromere; on chromosome X, the most proximal marker was at 33.5 Mb from the centromere. The most distal marker was within 5.8 Mb of the telomere for all chromosomes. Internal markers were no more than 24.6 Mb from each other (chromosome 1), except on chromosome 2, where two markers were spaced 64.4 Mb apart. Mean spacing was approximately 7.88 Mb between markers. Physical distances quoted in this paper were taken from mouse genome build 37.2, and SNP data were taken from dbSNP Build 128. DNA samples from each parental strain and one F_1_ mouse produced from each direction were used as control samples. Data-checking analyses suggested by Broman et al. [24] were run before mapping analysis to ensure the quality and integrity of both phenotype and genotype data. Due to inconsistencies in the genotyping results, an albino female mouse from the low avascular area group was excluded from subsequent analysis.

The genotype of the tyrosinase locus of the mice not selected for genotyping was inferred from coat color. In this cross, albinism was conferred by homozygosity for the BALB/c allele of tyrosinase. The genotype of albino mice at the tyrosinase locus was encoded as A^B/c^A^B/c^. For all non-albino mice, a code indicating “not-A^B/c^A^B/c^” was used.

### QTL mapping, identification of covariates

Coat color, sex, pigmentation and the strain of the paternal grandmother were tested using ANOVA (ANOVA) models to identify traits associated with the primary phenotypes of interest (weight and unadjusted avascular areas). The inclusion of covariates in mapping analyses reduces residual variation and enhances QTL detection [24]. Any variable found to have a statistically significant association was included in mapping models, as described below.

### Linkage analysis

The data were analyzed using R/qtl software [25]. Initial linkage analysis was performed using standard interval mapping to determine a logarithm of odds (LOD) score correlating the genotype with the phenotype for each marker. The weight and unadjusted avascular area were analyzed separately as primary phenotypes. Analyses were also performed using the sex, paternal grandmother, pigmentation, and weight as covariates. All covariates were compared as additive and interactive covariates in separate models to determine the best fit. To account for the multiple testing inherent in genome-wide QTL studies, and to control the type I error rate such that the probability of a false positive over all tests is below some value, significance thresholds were determined empirically via permutation testing [26]. We estimated the experiment-wise significance thresholds using 1,000 permutations for each data set, defining the LOD thresholds for highly-significant QTL (at p<0.05) and significant QTL (at p<0.1). One thousand permutation replicates has been shown to be an adequate number for estimating critical thresholds [26], and is the number routinely employed in QTL mapping studies.

### Gene–gene interactions

Secondary analyses included composite interval mapping to determine influence of markers near QTL on other loci (i.e., gene–gene interactions). Additionally, two dimensional, two-QTL scans were performed to consider linked or interacting QTL.

### Estimating QTL effects

To estimate the contribution of QTL identified in the linkage analysis, the allelic effects were calculated and compared between mice categorized by genotype (A^B/c^A^B/c^, A^B/c^A^C57^, A^C57^A^C57^). Additive and dominance effects were also considered in calculations. The QTL effects were assumed to correspond to the SNP marker nearest the peak LOD score.

## Results

### Differences in response to OIR between strains

**Figure 2:**
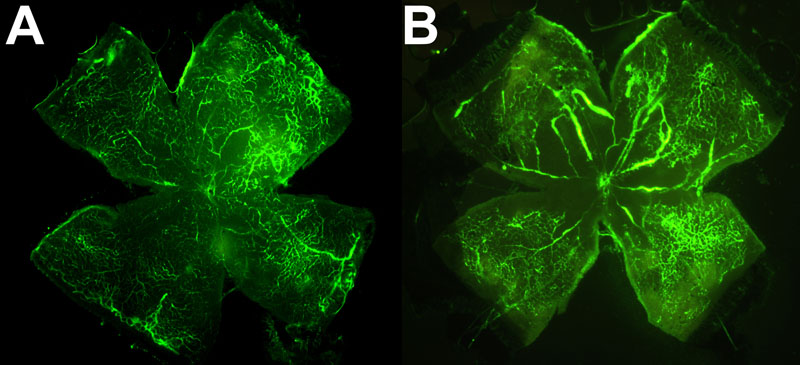
Retinas after oxygen induced retinopathy (OIR) protocol. **A**: Flatmounted retina of a BALB/cByJ mouse on postnatal day 16 after exposure to the OIR protocol. The retina is relatively well vascularized at this time point. **B**: A retina from a C57BL/6ByJ mouse at the same time point after exposure to the OIR protocol. The central retina has large areas of avascularity.

To confirm the response of parental strains to the OIR model, 15 BALB/cByJ offspring from three litters and ten C57BL/6ByJ mice from three litters were exposed to hyperoxia, as described. Typical flatmounted fluorescein dextran-perfused retinas are shown in Figure 2. The measurement of mean retinal avascular areas on P16 showed C57BL/6ByJ mice to have a 2.9 fold greater unadjusted area than had the BALB/cByJ mice (1.55 mm^2^ versus 0.53 mm^2^, Welch’s two-sample *t*-test, p<0.0005).

**Figure 3:**
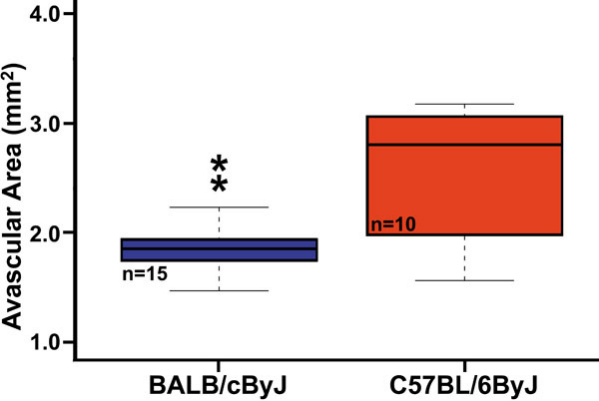
Mean avascular areas of parental strains after adjusting for influence of weight. This shows that the BALB/cByJ strain has smaller areas of avascular retina in response to hyperoxia than the C57BL/6ByJ strain (ANOVA, p=0.0002).

Weight ranged from 4.15 to 10.95 g. BALB/cByJ mice weighed more on P16 than did their C57BL/6ByJ counterparts (with means of 7.64 and 6.05 g, respectively, ANOVA, p=0.009). After adjusting avascular areas for weight, as described above, the C57BL/6ByJ mice were still found to have greater avascular areas than had BALB/cByJ mice (2.60 mm^2^ versus 1.86 mm^2^, ANOVA, p=0.0002, Figure 3), suggesting that the difference in avascular area was not due to differences in weight alone.

No differences in unadjusted avascular areas were found between male and female mice, either within each strain or across strains (ANOVA, BALB/cByJ, p=0.4, C57BL/6ByJ, p=0.5; Student’s *t*-test, all mice: p=0.06). The same was true when comparing adjusted avascular area between mouse genders (ANOVA, BALB/cByJ, p=0.07, C57BL/6ByJ, p=0.15; Student’s *t*-test, all mice: p=0.08).

### Effect of covariates on phenotypes in F_2_ population

On P16, weight was investigated as a primary phenotype to identify potential QTL related to body mass. ANOVA tests were performed to investigate the correlation of secondary phenotypes with weight, but no statistically significant association between weight and the sex, strain of the paternal grandmother, or coat color was found.

**Figure 4:**
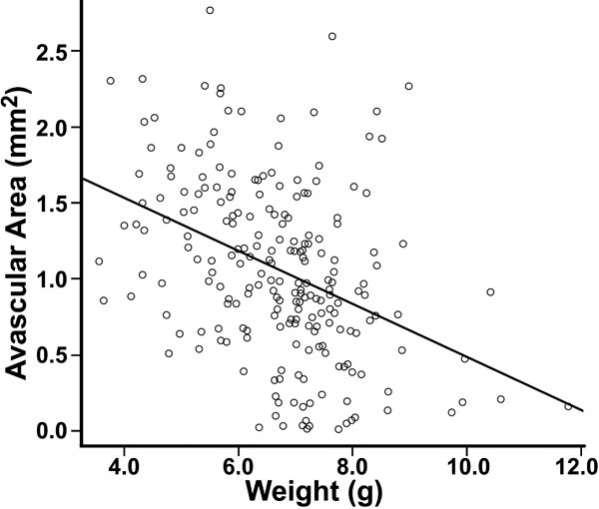
The inverse association between avascular area and weight of F_2_ mice at postnatal day 16 after exposure to oxygen-induced retinopathy. The avascular area was shown to correlate with weight with a coefficient of −0.1743. This association was shown to be statistically significant (Welch’s two-sample *t*-test, p<10^−8^).

F_2_ mice had unadjusted avascular areas ranging from 0.013 to 2.76 mm^2^, with a mean of 1.06 mm^2^. On P16, their mean weight was 6.70 g, with a range from 3.56 to 11.77 g. Linear regression analysis showed that the avascular area was inversely associated with weight (ANOVA, p<10^−8^, Figure 4).

**Figure 5:**
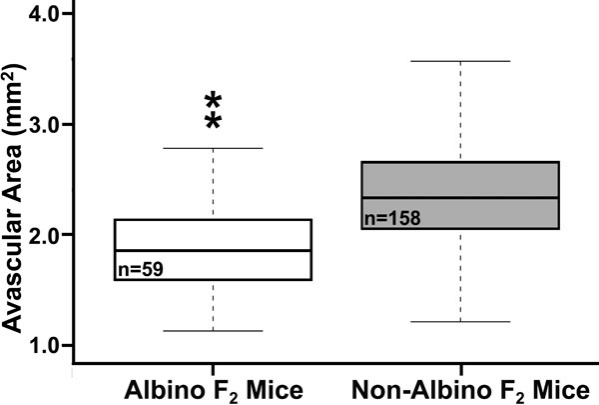
Comparison of avascular areas for albino and non-albino F_2_ mice. The mean avascular area of albino mice was found to be 0.67 mm^2^ versus 1.20 mm^2^ for non-albino mice, a statistically significant difference (ANOVA, p<0.0001).

Coat color pigmentation correlated with the adjusted retinal avascular area. Albino F_2_ mice exposed to the OIR model had smaller adjusted avascular areas than had pigmented mice (ANOVA, p<5×10^−8^, Figure 5). No associations between the avascular area and any of the individual non-albino coat colors were found (ANOVA, p=0.39).

The strain of the paternal grandmother was associated with an adjusted avascular area in the F_2_ generation (ANOVA, p=0.004). F_2_ mice produced from inbreeding (BALB/cByJ x C57BL/6ByJ)F_1_ parents had smaller avascular areas than had those produced from the reciprocal cross (2.12 mm^2^ versus 2.34 mm^2^). The paternal grandmother was included in the mapping analysis as an additive covariate. Segregating F_2_ mice by both the paternal grandmother and sex did not show any statistically significant differences (ANOVA, p=0.41), suggesting that the two covariates did not interact.

In the F_2_ mice, unlike in the parental generation, gender was associated with the avascular area (the avascular area was 2.16 mm^2^ for males, 2.28 mm^2^ for females, ANOVA, p=0.02). Gender was not found to interact with other secondary phenotypes such as weight, coat color, or the paternal grandmother (ANOVA, p=0.08, 0.52, and 0.40, respectively) in the F_2_ generation.

ANOVA testing of the weight, paternal grandmother, and sex did not show any statistically significant interaction between the covariates (p>0.05). Therefore, they were included as additive covariates in mapping analyses.

On P16, analyses were performed to identify QTL potentially related to body mass. ANOVA tests were run to consider the candidacy of secondary phenotypes as covariates, but no statistically significant associations between the weight and the sex, strain of the paternal grandmother, or coat color were found.

### Identification of QTL related to OIR response

#### Mapping without covariate inclusion

**Figure 6:**
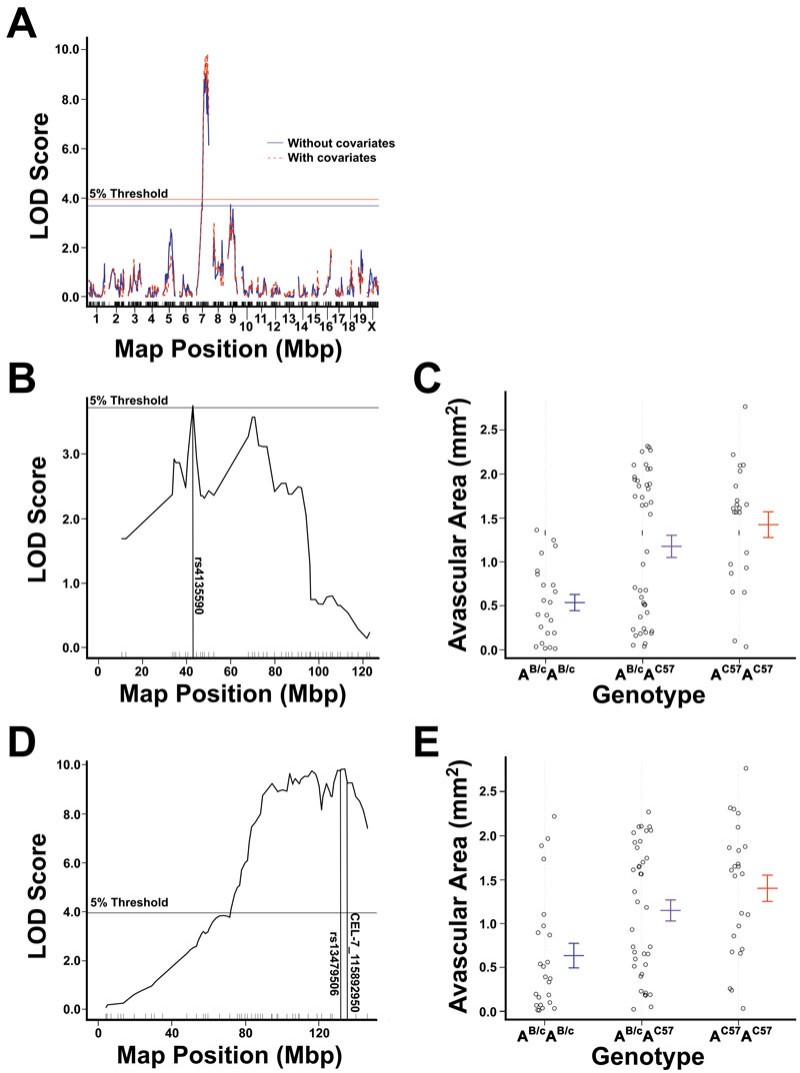
The results of genetic linkage mapping using central retinal avascular area as the phenotype. **A**: Linkage map of avascular area across the whole genome created using no covariates (blue) and using weight, paternal grandmother, and sex as additive covariates in the analysis (red). Empirically derived genome-wide (alpha=0.05) thresholds of significance are shown as horizontal lines. For mapping without covariates the threshold of significance was a logarithm of odds (LOD) score of 3.75. For mapping with covariates the threshold of significance LOD score of 3.81. Mapping without covariates identified peaks on chromosomes 7 (LOD=8.90) and 9 (LOD=3.75). Mapping with covariates identifed a peak on chromosome 7 (LOD=9.83). **B**: Linkage map of avascular area across chromosome 9 created without inclusion of covariates, showing a peak at SNP rs4135590 (LOD=3.75, p=0.05). **C**: Effect plots of primary peak on chromosome 9. The BALB/cByJ allelotype was found to be additively protective against the development of OIR. **D**: Linkage map of avascular area across chromosome 7 created by including weight, paternal grandmother, and sex as additive covariates, showing a peak between SNPs rs13479506 and CEL-7_115892950 (LOD=9.83, p<0.004). **E**: Effect plot of the peak on chromosome 7 at the nearest marker, CEL-7_115892950. The BALB/cByJ allelotype at this locus is shown to have a protective effect in a recessive manner.

The initial QTL analysis employed a covariate-free model. This analysis identified two LOD score peaks associated with avascular areas that reached the genome-wide threshold of α=0.05. These peaks occurred on chromosomes 7 and 9 (Table 1, Figure 6A). On chromosome 9, the peak LOD score (3.75, p=0.05, Figure 6B) occurred at SNP rs4135590, located 42.8 Mb distal from the centromere. The genotype at this QTL explained 6.5% of the variance in avascular areas (p=0.0007, Table 1), with each BALB/cByJ allele having an additive protective effect (Figure 6C). The 1.8-LOD support interval was found to be 12.4–96.0 Mb distal from the centromere.

**Table 1:** QTL associated with retinal avascular area in oxygen induced retinopathy

**Significance**	**Chromosome**	**Phenotype**	**LOD Score**	**Markers at or flanking QTL**	**Marker position (Mb)**	**LOD support interval (Mb)**	**Effect on Phenotype**
p<0.004	7	Avascular area (P16)	8.90^†^	rs13479427	107.2 131.8	81.2 – 146.5	0.175
			9.83*	rs13479506 CEL-7_115892950‡	135.8	88.2 – 146.5	0.342
p=0.05	9	Avascular area (P16)	3.75^†^	rs4135590	42.8	12.4 – 96.0	0.065
p=0.05	5	Weight (P16)	3.91^†^	CEL-5_93945748	97.1	52.8 – 133.6	0.041

† mapping analysis excluded covariates; * mapping analysis included weight, sex, and paternal grandmother as covariates. ‡ the two markers flanking the LOD score peak are reported.

The peak on chromosome 7 was located at SNP rs13479427, 107.2 Mb distal from the centromere. The LOD score of this peak was 8.90 (p<0.004). The QTL on chromosome 7 explained 17.5% of the phenotypic variance (Table 1, p<0.004). At this locus, the BALB/cByJ allele had a recessive protective effect. A 1.8-LOD support interval was determined to lie 81.2–146.5 Mbp distal from the centromere. The marker at 146.5 Mbp was the most telomeric marker included in the SNP panel, therefore we are unable to exclude the possibility that the support interval extends as far as the telomere.

A two-QTL model including the peaks on chromosome 7 and 9 found that these loci explained 23.2% of the phenotypic variance (p=1.8×10^−11^), with a combined LOD score of 12.4.

#### Mapping with covariates

Using weight, the strain of the paternal grandmother, and sex as additive covariates, a single LOD score peak associated with the unadjusted avascular area was identified on chromosome 7 at a genome-wide threshold of α=0.05 (Table 1, Figure 6D). This QTL is located at 134.2 Mb, between SNPs rs13479506 and CEL-7_115892950. The LOD score of this peak was 9.83 (p<0.004). The QTL on chromosome 7, in conjunction with the covariates, explained 34.2% of the phenotypic variance (Table 1, p<0.004). At this locus, the BALB/cByJ allele had an additive protective effect (Figure 6E). The 1.8-LOD support interval was calculated to extend from 88.2 to 146.2 Mb distal from the centromere. The distal boundary of this interval coincided with the most distal marker tested. Therefore, it is possible that if a marker at the telomere had been tested, it would have been found that the interval extended as far as the telomere.

### Locus–locus interactions

Composite interval mapping was conducted using the genotype at the putative QTL on chromosome 7 as an additive or interactive covariate with other loci; this model reduced residual variation to clarify evidence for further QTL, allowing detection of background loci with weak main effects [24]. No additional loci were discovered. A two-dimensional, two-QTL scan was conducted to identify any epistatic interactions. No statistically significant linked or multiple QTL were found.

### Identification of a QTL related to weight

**Figure 7:**
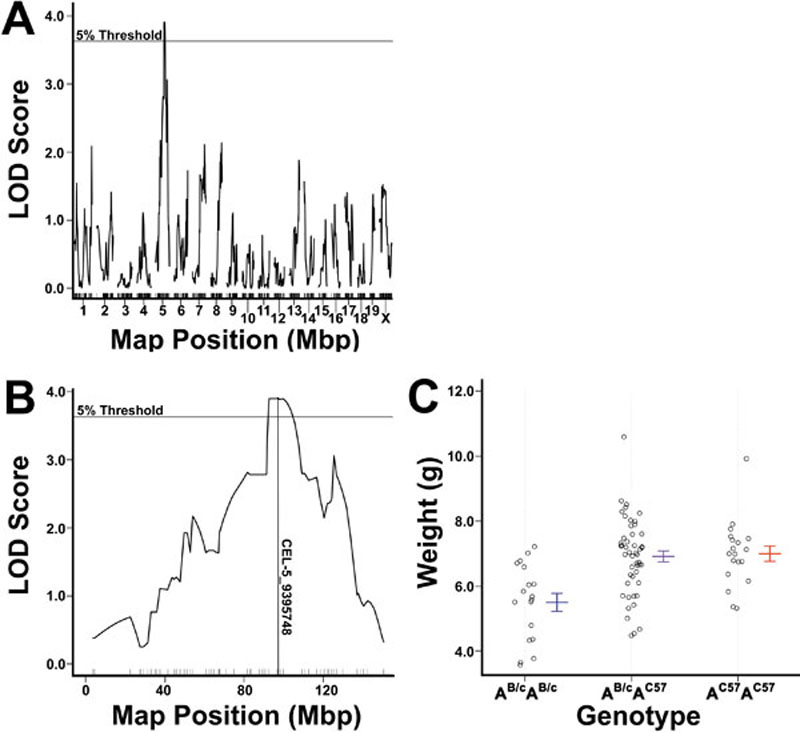
The results of genetic linkage mapping using bodyweight 96 h after exposure to hyperoxia as the phenotype. **A**: A genome-wide linkage map of weight on postnatal day 16 after exposure to the oxygen-induced retinopathy model. The threshold of significance was found to be 3.86 (α=0.05), as determined by permutation tests. **B**: Shows linkage across chromosome 5, with significant linkage at marker CEl-5_93945748 (LOD=3.91, p=0.024). **C**: Effect plot of genotypes at this marker on postnatal day 16; homozygosity for the BALB/cBJ allelotype at this locus is associated with reduced weight in a recessive manner (ANOVA, p<0.001). The BALB/cByJ allele is designated A^B/c^, and the C57BL/6ByJ alleles is designated A^C57^.

Standard interval mapping identified an LOD score peak on chromosome 5 that reached the genome-wide threshold for high significance (α=0.05, as determined by permutation tests). The peak occurred at the SNP CEL-5_9395748, approximately 97.1 Mb distal from the centromere (LOD=3.91, p=0.024, Figure 7). On P16, at this locus, the C57BL/6ByJ allele was found to be associated with greater weight in a dominant fashion. Using this single-QTL model, the genotype at this locus was estimated to explain 4.1% of the variance in weight (p=0.01). A 1.8-LOD support interval was derived using an algorithm in R/qtl by interpolating between markers on the basis of recombination frequency, and was found to be 52.8–133.6 Mb distal from the centromere.

### Identification of candidate genes

A list of candidate genes located within the 1.8-LOD support intervals for the QTL was created using a web-based virtual positional cloning program, Positional Medline Database [27], queried March 15, 2011. The program identifies candidate genes for the LOD support interval of putative QTL related to keywords. The search region on chromosome 7 was extended to the telomere (152 Mb); 160 candidate genes were identified in this interval using “retina AND angiogenesis” as keywords in the query. The ten genes most-correlated with the search terms were mitogen activated protein kinase 3 (*Mapk3*), antigen KI-67 (*Mki67*), fibroblast growth factor 4 (*Fgf4*), fibroblast growth factor receptor 2 (*Fgfr2*), insulin-like growth factor 2 (*Igf2*), tyrosine hydroxylase (*Th*), fibroblast growth factor 3 (*Fgf3*), extra cellular link domain-containing 1 (*Xlkd1*), BCL2/adenovirus E1B interacting protein 3 (*Bnip3*), and cyclin D1 (*Ccnd1*). Using “oxidative stress” as keywords in the query over the same interval to identify potential candidate genes related specifically to vaso-obliteration, Positional Medline identified 231 genes; the top ten included: *Mapk3, Th, Mki67,* cytochrome P450 2E1 (*Cyp2e1*), amyloid beta A4 (*Apbb1*), parathyroid hormone (*Pth*), integrin alpha M (*Itgam*), integrin alpha L (*Itgal*), integrin-linked kinase (*Ilk*), and *Igf2*. Twenty-five genes were found within the support interval of the QTL on chromosome 9 using “retina AND angiogenesis,” including alpha disintegrin and metallopeptidase domain 10 (*Adam10*), chondroitin sulfate proteoglycan 4 (*Cspg4*), alpha crystallin B (*Cryab*), sphingosine-1-phosphate receptor 2 (*Edg5*), Pbx/knotted 1 homeobox 2 (*Pknox2*), erythropoietin receptor (*Epor*), dopamine receptor 2 (*Drd2*), heat shock protein 2 (*Hspa2*), cytochrome P450 family 1 subfamily a polypeptide 2 (*Hspb2*), cytochrome P450 family 1 subfamily a polypeptide 2 (*Cyp1a2*), and cytochrome P450 family 1 subfamily a polypeptide 1 (*Cyp1a1*); “oxidative stress” identified 246 candidates, including kelch-like ECH-associated protein 1 (*Keap1*), apolipoprotein A-I (*Apoa1*), beta-site APP cleaving enzyme 1 (*Bace1*), *Drd2,* low density lipoprotein receptor (*Ldlr*), intercellular adhesion molecule 1 (*Icam1*), cytochrome P450 family 19 subfamily a polypeptide 1 (*Cyp19a1*), amyloid beta (A4) precursor-like protein 2 (*Aplp2*), *Cyp1a2* and *Cyp1a1.* For the support interval found on chromosome 5 using weight as the primary phenotype, “birth weight” was used as the keyword in the query to derive 101 candidate genes, including scavenger receptor class B, member 1 (*Scarb1*), alpha fetoprotein (*Afp*), group specific component (*Gc*), chemokine (C-X-C motif) ligand 2 (*Cxcl2*), heparanase (*Hpse*), chemokine (C-X-C motif) ligand 1 (*Cxcl1*), casein beta (*Csn2*), amphiregulin (*Areg*), platelet factor 4 (*Pf4* or *Cxcl4*), and chemokine (C-X-C motif) ligand 10 (*Cxcl10*).

## Discussion

ROP remains a significant cause of vision loss in the pediatric population. Although some risk factors have been identified, its development is complex and involves both environmental and genetic factors. This study aimed to initiate the identification of genes that may play a role in ROP susceptibility. Using the murine OIR model, we developed a mapping cross between C57BL/6ByJ and BALB/cByJ inbred mouse strains. These mice were confirmed to have significantly different vascular responses to OIR on P16, with the BALB/cByJ strain having smaller avascular areas than the C57BL/6ByJ strain. These results support those published by Dorrell, et al. [20], which found that while both strains have similar vaso-obliterative areas upon their return to normoxia on P12, the BALB/cByJ strain revascularizes more rapidly than the C57BL/6J strain.

Genetically determined differences in retinal ocular morphology between the two strains may play a role in their responses to OIR. Recent reports using confocal scanning laser ophthalmoloscopy confirm physiologic differences in retinal structure between adult C57BL/6J and BALB/c mice, with BALB/c mice having significantly thinner central retinas (211 μm versus 237 μm) [28]. The differences between C57BL/6J and BALB/c mice are at least partially related to C57BL/6J being a pigmented strain, while BALB/c is an albino strain [29–31], and this issue is discussed further below.

The mapping cross revealed QTL associated with susceptibility to OIR on chromosomes 7 and 9. The BALB/cByJ allele of the chromosome 7 QTL was found to be protective in a recessive manner. In a mapping model that includes weight, sex, and the paternal grandmother as additive covariates, the allelotype at this marker is estimated to explain 34% of the phenotypic variation. The BALB/cByJ allele of the chromosome 9 QTL was also found to be protective in a recessive manner; this peak only reached statistical significance when covariates were excluded from the model. In a single QTL model, this allele was estimated to explain 7% of the variation in phenotype.

No QTL was identified on the X chromosome, despite evidence that the strain of the paternal grandmother was associated with the avascular area. It was noted that when initially performing mapping analyses with single covariates to confirm their inclusion in the final model, the inclusion of the paternal grandmother alone caused a loss of the peak on chromosome 9. This suggests that the directionality of the cross may influence susceptibility via an epigenetic effect on a gene or genes within this locus, rather than through a modifier gene on the X chromosome.

A QTL related to weight on P16 was identified on chromosome 5. The C57BL/6ByJ allele was found to be associated with greater weight in a dominant manner; the genotype at this locus was estimated to explain 4.1% of the variance in weight at this time point. The selective genotyping model based on avascular area, rather than weight, may have introduced bias in estimating the effect of this QTL on weight and avascular area. In both human ROP and murine OIR, increased birthweight and subsequent weight gain are protective against neovascularization [32,33]; although C57BL/6ByJ mice had larger avascular areas on P16 than had their BALB/cByJ counterparts, the data suggest that the C57BL/6ByJ genome does contain an allele related to weight that serves to protect against OIR.

The initial analysis of F_2_ mice revealed significantly different retinal avascular areas in albino and non-albino mice. This result supports findings from a rat backcross [17] as well as across different strains of rats [34]. Because albinism in this cross is caused by the homozygous loss of function of tyrosinase, the rate-limiting enzyme in the biosynthesis of melanin [31], these data suggest that tyrosinase or a linked gene might play a role in disease susceptibility. The QTL identified on chromosome 7 by mapping analysis includes tyrosinase (located at 94.58–94.64 Mb) within the 1.8-LOD support interval. An intriguing possibility is that tyrosinase itself is a modifier of the area of the avascular retina in rodent OIR.

The tyrosinase gene has various roles in ocular physiology. There are numerous morphological differences between the retinas of albino and non-albino mice, and it is not clear whether these may be associated with responses to hyperoxia. It is possible that the thicker C57BL/6 retina becomes more hypoxic than the BALB/c retina, and that this leads to greater astrocyte degeneration and slower revascularization [29–31]. The absence of melanin in the retinal pigment epithelium may alter the response of the retina to oxidative stress [17]. Alternatively, since the absence of melanin in the iris increases the amount of light reaching the retina [35], it is possible that greater retinal light exposure affects the revascularization process, even though light has not previously been demonstrated to play a role in modifying the severity of OIR [36]. Furthermore, tyrosinase can form L-3,4-dihydroxyphenylalanine (L-dopa), the immediate precursor of dopamine [37]. The role of dopamine metabolism in OIR is currently unknown, but dopamine is known to modulate tumor angiogenesis [38], and may play a role in angiogenesis in OIR. Since albinism interacts with retinal physiology in many ways, we are performing further experiments to investigate the candidacy of tyrosinase. However, neither our experiment nor the work that found an association between albinism and OIR in rats [17] were designed to test the candidacy of any particular gene.

Positional Medline identified several other interesting candidate genes, located within the support interval of the QTL on chromosome 7, using the keywords “retina AND angiogenesis.” MAPK3, or ERK-1, is a mitogen-activated kinase that is activated by fibroblast growth factor (FGF) to induce endothelial cell proliferation in retinal angiogenesis [39], and has been shown to play a role in regulating VEGF expression in response to oxidative stress [40]. FGF4, a member of the FGF family, is a mitogen that is involved in the development and morphogenesis of various cell and tissue types [41]. Overexpression of FGFR2, a receptor for FGF, has been demonstrated in several types of neoplasia, including breast cancer [42], prostate cancer [42], renal cell carcinoma [43], and gastric cancer [44]. Within the retina, insulin-like growth factor 2 is present almost exclusively in the photoreceptors and vasculature, where it is the major locally expressed growth factor. However, it is not expressed in the vascular endothelial cells in the neovascular tufts formed in response to OIR [45]. Tyrosine hydroxylase is the enzyme primarily responsible for dopamine production in the central nervous system and retina; in the eye, it is found primarily in amacrine cells in the inner plexiform layer [46]. *Xlkd1*, or extra-cellular link domain containing 1, encodes a hyaluronan receptor expressed on the lymphatic endothelium in both adult and embryonic vessels, and XLKD1-positive cells were recently identified in the hyaloid vascular system of the developing murine eye [47].

Four of the top ten putative genes identified by using the key terms “oxidative stress” (*Mapk3, Th, Mki67,* and *Igf2*) were also identified in the prior search. Integrin alphas L, and M (CD11a and b, respectively) are expressed on neutrophils, and when complexed with beta-2 integrin CD18, function to modulate the migration of the cells to the site of the injury by binding intracellular adhesion molecule 1 (ICAM1) on endothelial cells [48]. VEGF-A has been shown to stimulate neutrophil and T-cell recruitment in an ITGAL- and ITGAM-dependent manner [49]. Integrin-linked kinase (ILK)-signaling has been shown to mediate the production of the brain-derived neurotrophic factor by endothelial cells [50], and oxidative stress disrupts this signaling pathway [51].

Although ranked less highly by the Positional Medline algorithm, three genes related to Wnt signaling pathways were included in the list of candidate genes. Wnt signaling plays an essential role in cell survival, proliferation, and migration, and has an important role in controlling both developmental retinal angiogenesis and retinal neovascularization in disease states [52]. Mutations in genes related to this pathway have been implicated in several ocular neovascular diseases with phenotypic similarities to ROP, including familial exudative vitreoretinopathy and Norrie disease [53]. Frizzled-4 (FZD4) is a Wnt receptor that functions in both canonical and noncanonical signaling pathways. Mutations in FZD4 have been identified as a cause of familial exudative vitreoretinopathy [54] and have been confirmed in cases worldwide [55,56]. More recently, FZD4 mutations have been found in a small number (3%) of a cohort of infants with severe ROP [11]. Interestingly, the mouse *Fzd4* gene lies within the QTL found on chromosome 7. *Wnt11* is another gene involved in Wnt signaling that is encoded within the QTL on chromosome 7; WNT11 is a noncanonical ligand in the pathway. Within the retina specifically, mutations in *Wnt11* have been shown to result in increased angiogenesis and branching [57]. Dickkopf 3 (DKK3) belongs to a family of secreted glycoproteins that act as regulators of Wnt signaling. In the retina, *Dkk3* is expressed by Müller glia and retinal ganglion cells, and is a cell type-specific positive regulator of Wnt signaling. In response to photoreceptor death in a murine model of retinal degeneration, *Dkk3* expression was upregulated, and functioned to inhibit caspase activation in Müller glia [58].

The LOD support interval on chromosome 9 also contains several interesting candidates identified by Positional Medline, related to the retina and angiogenesis. Chondroitin sulfate proteoglycan 4 (CSPG4), also known as NG2, is expressed by nascent pericytes early in angiogenesis. Studies of ischemic retinal neovascularization have shown decreased ectopic vessel protrusion into the vitreous, and decreased proliferation of both pericytes and endothelial cells in NG2 knockout (KO) mice [59]. Similarly to MAPK3, MAP2K1 (MEK1) mediates FGF-stimulated endothelial-cell proliferation in tumor models [39]. IL18 has been shown to play a role in regulating the regression of pathological neovascularization in mice after their exposure to the OIR model [60]; additional studies using KO mice suggest that IL18 plays a role in regulation of retinal vessel formation [61]. Crystalline alpha B is a chaperone within the small heat-shock protein family and has been shown, using KO mouse models, to play a role in intraocular angiogenesis by acting as a chaperone for VEGF-A [62]. Erythropoietin (EPO) has been linked to both ROP and diabetic retinopathy. Local tissue EPO levels are suppressed during retinal vessel loss in OIR and elevated during neovascularization, and the pharmacological use of EPO has been shown to protect against hypoxia-induced apoptosis [63]. The siRNA inhibition of EPO expression during the neovascular phase of OIR has been shown to inhibit neovascularization [64]. Human and murine studies have both found that vitreal EPO expression is associated with proliferative diabetic retinopathy [65].

Candidates derived using the key terms “oxidative stress” included several adhesion molecules. ICAM1 is expressed by endothelial cells and plays a critical role in leukocyte stasis and migration to the injury site [66]. Dopamine receptor 2 acts as an antioxidant at physiologic concentrations of dopamine; it has been found to regulate the production of reactive oxidative species, and KO mice have higher blood pressure in part due to the increased oxidative damage in the kidney [67]. The low-density lipoprotein receptor is also involved in mediating oxidative stress; KO mouse models have increased oxidative stress, inflammation, and atherosclerosis [68]. Furthermore, oxidative modification of this receptor has been associated with endothelial cell dysfunction [69]. Three different cytochrome P450 genes were also found to be candidates by the Positional Medline program; however, this may be a spurious result due to the large amount of data published regarding oxidative stress generated in the metabolism of various drugs.

The support interval of the QTL on chromosome 5 associated with weight includes 101 candidate genes related to “birth weight.” Low maternal serum α-fetoprotein (AFP) levels have been shown in humans to be associated with high birthweight infants [70]. Heparanase, HPSE, plays a role in placental development and affects both structure and nutrient transport [71]. CXCL1, 2, 4, and 10 are chemokines that have been studied extensively in regard to their role in inflammation [72,73], development [73], angiogenesis [73,74], and neoplasia [74].

In conclusion, revascularization in OIR is related to the weight, strain of paternal grandmother, sex, and albinism. Our data support the existence of a QTL on chromosome 5 that influences weight after exposure to hyperoxia, as well as QTL on chromosomes 7 and 9 that modify susceptibility to OIR. Ongoing studies are investigating the role of candidate quantitative trait genes as modifiers of the progression of OIR. It is hoped that investigation of quantitative trait genes will help to elucidate the pathogenesis of ROP. These modifier genes may also be candidates for susceptibility genes in other complex ocular diseases involving aberrant angiogenic responses, such as diabetic retinopathy and neovascular age-related macular degeneration.
